# Glucose Metabolism as a Pre-clinical Biomarker for the Golden Retriever Model of Duchenne Muscular Dystrophy

**DOI:** 10.1007/s11307-018-1174-2

**Published:** 2018-03-05

**Authors:** Sarah Morar Schneider, Vidya Sridhar, Amanda K. Bettis, Heather Heath-Barnett, Cynthia J. Balog-Alvarez, Lee-Jae Guo, Rachel Johnson, Scott Jaques, Stanislav Vitha, Alan C. Glowcwski, Joe N. Kornegay, Peter P. Nghiem

**Affiliations:** 1 Department of Veterinary Pathobiology, College of Veterinary Medicine and Biomedical Sciences, Texas A&M University, College Station, TX 77843-4458 USA; 2 Texas A&M Institute for Preclinical Studies, College of Veterinary Medicine and Biomedical Sciences, Texas A&M University, College Station, TX 77843-4458 USA; 3 Department of Veterinary Integrative Biosciences, College of Veterinary Medicine and Biomedical Sciences, Texas A&M University, 4458 TAMU, College Station, TX 77843-4458 USA; 4 Texas A&M Veterinary Diagnostic Laboratory, College of Veterinary Medicine and Biomedical Sciences, Texas A&M University, College Station, TX 77843-4458 USA; 5 Microscopy Imaging Center, College of Veterinary Medicine and Biomedical Sciences, Texas A&M University, College Station, TX 77843-4458 USA

**Keywords:** GLUT4, GRMD, DMD, Dog, Glucose, Insulin, Metabolism, PET/CT, Biomarkers, Dystrophin

## Abstract

**Purpose:**

Metabolic dysfunction in Duchenne muscular dystrophy (DMD) is characterized by reduced glycolytic and oxidative enzymes, decreased and abnormal mitochondria, decreased ATP, and increased oxidative stress. We analyzed glucose metabolism as a potential disease biomarker in the genetically homologous golden retriever muscular dystrophy (GRMD) dog with molecular, biochemical, and *in vivo* imaging.

**Procedures:**

Pelvic limb skeletal muscle and left ventricle tissue from the heart were analyzed by mRNA profiling, qPCR, western blotting, and immunofluorescence microscopy for the primary glucose transporter (GLUT4). Physiologic glucose handling was measured by fasting glucose tolerance test (GTT), insulin levels, and skeletal and cardiac positron emission tomography/X-ray computed tomography (PET/CT) using the glucose analog 2-deoxy-2-[^18^F]fluoro-d-glucose ([^18^F]FDG).

**Results:**

MRNA profiles showed decreased GLUT4 in the cranial sartorius (CS), vastus lateralis (VL), and long digital extensor (LDE) of GRMD *vs.* normal dogs. QPCR confirmed GLUT4 downregulation but increased hexokinase-1. GLUT4 protein levels were not different in the CS, VL, or left ventricle but increased in the LDE of GRMD *vs.* normal. Microscopy revealed diffuse membrane expression of GLUT4 in GRMD skeletal but not cardiac muscle. GTT showed higher basal glucose and insulin in GRMD but rapid tissue glucose uptake at 5 min post-dextrose injection in GRMD *vs.* normal/carrier dogs. PET/ CT with [^18^F]FDG and simultaneous insulin stimulation showed a significant increase (*p* = 0.03) in mean standard uptake values (SUV) in GRMD skeletal muscle but not pelvic fat at 5 min post-[^18^F]FDG /insulin injection. Conversely, mean cardiac SUV was lower in GRMD than carrier/normal (*p* < 0.01).

**Conclusions:**

Altered glucose metabolism in skeletal and cardiac muscle of GRMD dogs can be monitored with molecular, biochemical, and *in vivo* imaging studies and potentially utilized as a biomarker for disease progression and therapeutic response.

**Electronic supplementary material:**

The online version of this article (10.1007/s11307-018-1174-2) contains supplementary material, which is available to authorized users.

## Introduction

Duchenne muscular dystrophy (DMD) is a muscle wasting disease in boys due to loss of dystrophin [1, 2]. Membrane fragility causes myofiber degeneration, leading to loss of ambulation in adolescence and eventual death from cardiorespiratory complications [3]. In addition to, or as a consequence of, membrane fragility, dystrophic muscle is under increased metabolic stress due to decreased glycolytic/oxidative enzyme expression. There are also lower mitochondrial numbers, including some with abnormal structure, decreased intracellular ATP, and increased reactive oxygen species [4–9]. These alterations in dystrophic muscle lead to a “metabolic crisis” and a reduced capacity to respond to metabolic demands such as muscle contraction.

The main glucose transporter in cardiac and skeletal muscle [10], glucose transporter-4 (GLUT4), is abnormally located in subcellular aggregates in DMD [11]. In contrast, GLUT4 was found in membrane preps of a subcellular fractionation study in the mdx mouse model for DMD, suggesting a compensatory mechanism [11, 12]. GLUT4 translocation is controlled by both insulin and muscle contraction, which act through independent molecular pathways to increase cytoplasmic vesicle translocation to the cell membrane. GLUT4 transports back to the cytoplasm after insulin stimulation or contraction are withdrawn [13]. Since different vesicle pools of GLUT4 respond to insulin and contraction [14, 15], these two pathways may be cumulative. The membrane localization of GLUT4 in mdx mice may have functional significance, because dystrophic myofibers have an altered response to insulin [11, 14, 16].

Dogs with golden retriever muscular dystrophy (GRMD) have absent dystrophin [17, 18] and a more severe phenotype compared with mdx mice, suggesting that pre-clinical studies in affected dogs may better mirror anticipated outcomes in DMD. Specifically, GRMD dogs show a similar metabolic crisis in dystrophic muscle, with reduced glycolytic enzymes, decreased and abnormal mitochondria, and dysregulated AMPK expression [4, 19]. We hypothesized that GRMD myofibers would have altered GLUT4 localization (similar to DMD muscle) and subsequently altered (reduced) glucose metabolism. Based on our initial findings, GLUT4 was surprisingly localized to the sarcolemmal membrane, which led to rapid clearance of blood glucose and increased muscle uptake of a glucose analog. Our results suggest that glucose metabolism can be monitored and potentially utilized as a pre-clinical biomarker in GRMD dogs.

## Materials and Methods

Materials and methods are provided in the electronic supplementary material (ESM).

## Results

### MRNA Profiling and qPCR

**Fig. 1:**
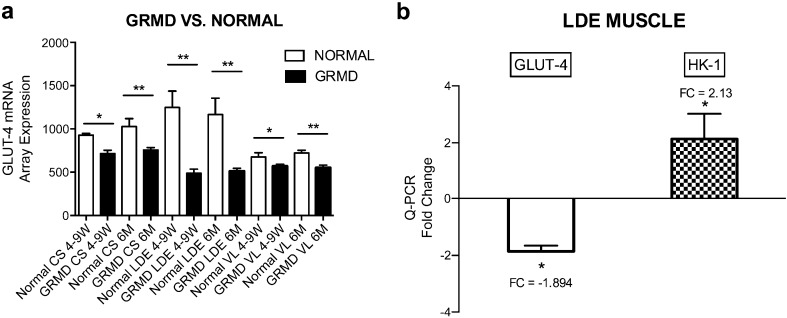
. GLUT4 mRNA expression was reduced in GRMD *vs.* normal muscle. **a** GLUT4 mRNA expression was reduced in the CS, VL, and most pronounced in the LDE (50 % decrease) of GRMD (black bars) compared with normal (open bars) dogs at 4–9 weeks and 6 months. **b** QPCR for GLUT4 on GRMD LDE muscle at 6 months confirmed downregulation by a fold change of − 1.894, while hexokinase 1 (HK-1) was upregulated by a fold change of + 2.13. *p < 0.05; **p < 0.01.

GLUT-4 expression from genome-wide mRNA profiles [20, 21] of GLUT4 mRNA in the CS, LDE, and VL muscles of GRMD was decreased at 4–9 weeks and 6 months compared with normal, with differences being most pronounced in the LDE (Fig. 1a). Specifically, GLUT4 mRNA expression was approximately 50 % less in GRMD LDE compared with normal, while the decrease in the CS and VL was only ~ 10–25 %. The LDE in normal dogs had higher basal GLUT4 expression compared with the CS and VL. QPCR for GLUT4 in the GRMD LDE at 6 months confirmed its downregulation by a fold change of − 1.894 (standard error of ± 0.20028; *p* = .0245) (Fig. 1b). Interestingly, in the same set of GRMD LDE muscle, Hexokinase-1 (HK1) was upregulated by a fold change of + 2.13 (standard error ± 0.88863; *p* = .046) (Fig. 1b).

### Western Blots

**Fig. 2:**
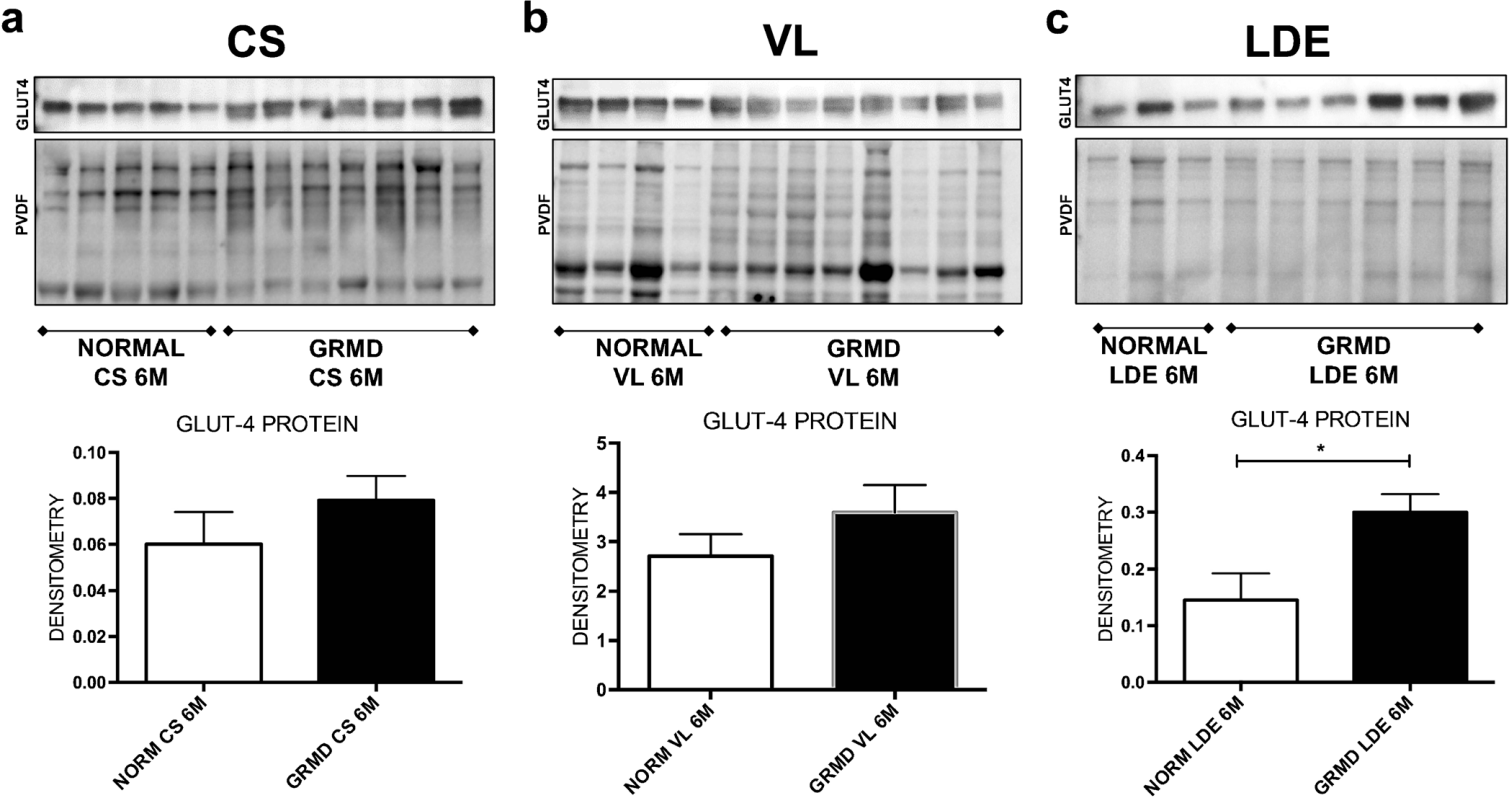
GLUT4 protein in GRMD *vs.* normal muscles. GLUT4 expression was similar in **a** CS and **b** VL between GRMD (black bars) and normal (white) dogs, but increased in **c** GRMD LDE at 6 months. GLUT4 expression was normalized to total protein/lane on the PVDF membrane. *p < 0.05.

GLUT4 protein expression did not significantly differ between normal and GRMD CS, VL, or left ventricle muscles (Fig. 2). GLUT4 protein expression at 6 months was significantly increased (*p* < 0.05) in GRMD LDE muscle, being nearly twice that of normal dogs.

### Confocal Microscopy

**Fig. 3:**
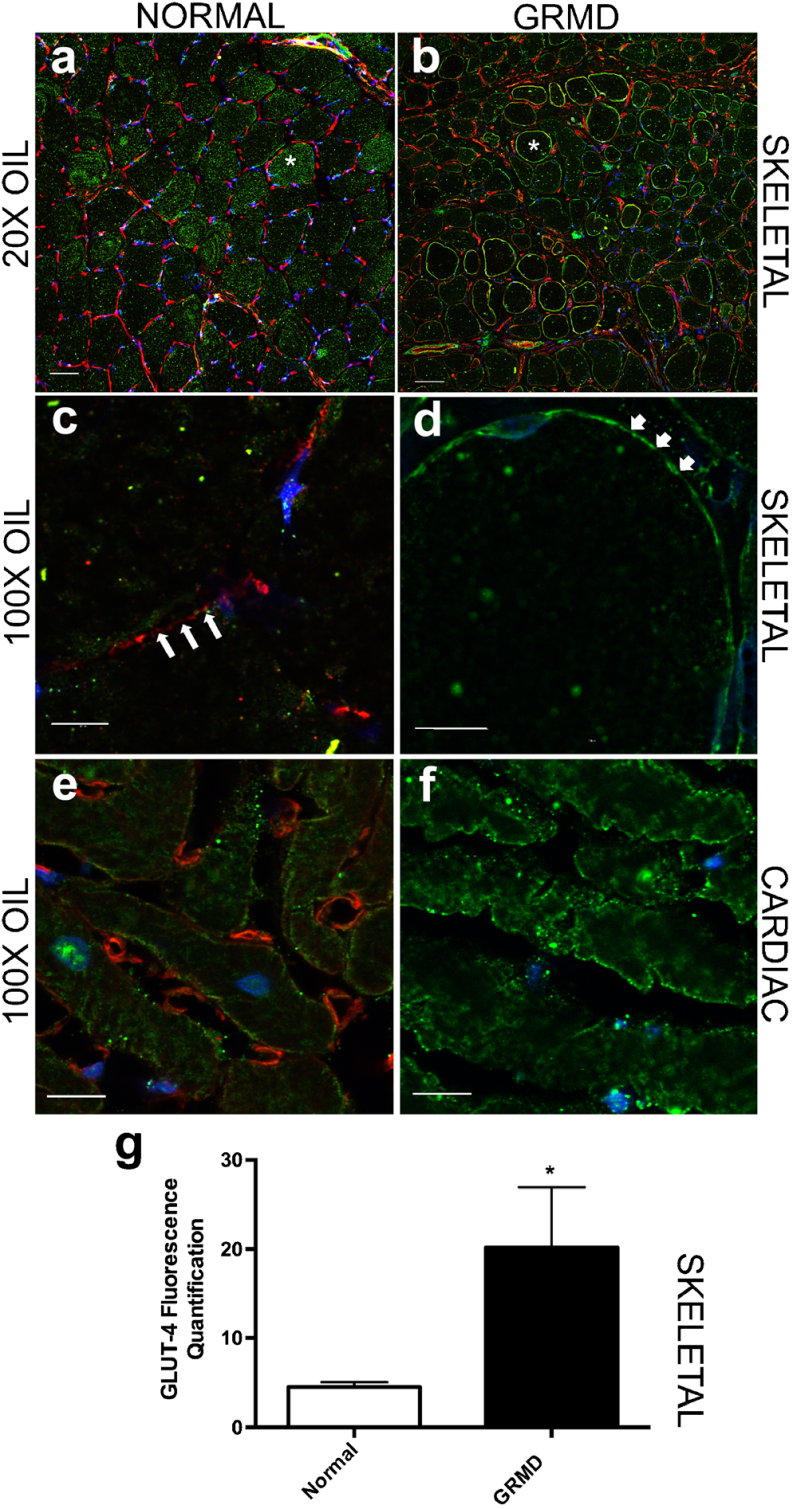
Peri-membranous localization of GLUT4 in GRMD skeletal muscle. Confocal microscopy showed increased localization of GLUT4 (green) at the myofiber membrane (white asterisk and arrows) in **b**, **d** GRMD skeletal muscle (CS, LDE, VL, cranial tibial, diaphragm), compared with **a**, **c** normal. Mainly, cytoplasmic GLUT4 localization was observed in normal muscle (asterisk; **a**, **c**). Note that GRMD also demonstrated cytoplasmic GLUT4 aggregates. In cardiomyocytes, there was no statistical difference in GLUT-4 membrane expression between **e** normal and **f** GRMD. **g** Quantification showed a nearly fourfold increase of GLUT4 at the membrane in GRMD skeletal muscle (*p* = 0.047); **a**, **b** × 20 objective (oil immersion); **c**–**f** × 100 objective (oil immersion). Nuclei, blue; spectrin, red. *N* = 5–6 per group for skeletal muscle; *N* = 3 per group for cardiac muscle.

GLUT4 was diffusely localized to the myofiber membrane in all GRMD skeletal muscles evaluated (CS, LDE, VL, cranial tibial, and diaphragm; *p* = .047; Fig. 3b, d, and g), whereas normal muscle showed minimal membrane localization (Fig. 3a, c). Cytoplasmic GLUT4 aggregates were present in both GRMD and normal muscle (Fig. 3a-d). There was no difference in membrane expression between normal and GRMD left ventricle muscles from the heart (Fig. 3e, f).

### Intravenous Glucose Tolerance Test

**Fig. 4:**
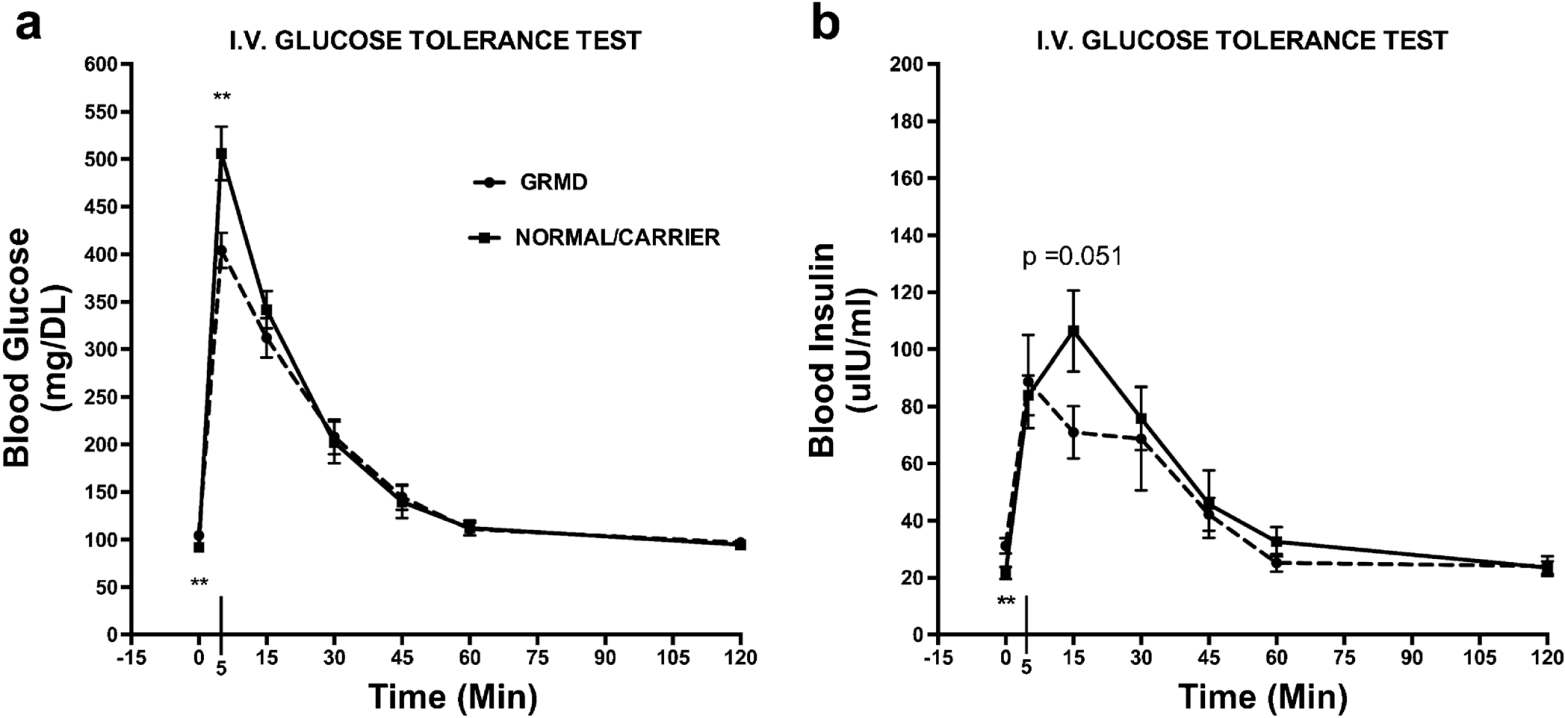
Rapid dextrose uptake in GRMD with an intravenous glucose tolerance test. At early time points, **a** glucose and **b** insulin curves were significantly different between GRMD and carrier/normal dogs. Resting (basal, time = 0) blood glucose was significantly higher in GRMD (104.5 mg/dl ± 7.3) compared with normal/carrier dogs (92.0 mg/dl ± 8.9). Dextrose uptake was rapid in GRMD dogs compared with normal at 5 min post-injection. Glucose levels were similar between genotypes at later time points. Resting (basal) insulin levels were significantly higher in GRMD dogs (31.26 μIU/ml ± 6.1; 35 % increase) *vs.* normal and carrier dogs (21.61 μIU/ml ± 7.27) and GRMD insulin peaked at the 5-min time point compared with 15 min in carrier/normal dogs. Insulin levels were similar between genotypes at later time points. **p < 0.01.

Based on the increased skeletal muscle membrane localization of GLUT4 demonstrated on confocal microscopy, we hypothesized that GRMD dogs would have more rapid clearance of plasma glucose and performed an intravenous glucose tolerance test (IV-GTT). For blood glucose (BG) values, both genotype (*p* = 0.002) and the interaction of time and genotype (*p* = 0.003) were significant. GRMD had higher basal BG levels compared with normal/carrier dogs (time 0; Fig. 4a). At 5 min post-dextrose injection, there was rapid tissue uptake of glucose from the blood in GRMD (mean 507 mg/dl) compared with carriers (mean 644 mg/dl; *p* < 0.05) and normal dogs (mean 597 mg/dl; *p* < 0.05) (Fig. 4a). Normal and carrier BG values were not significantly different.

### Insulin Curve

With regard to the IV-GTT, insulin values were also significantly affected by genotype (*p* = 0.007), although the interaction with time was not significant. There was a 45 % increase in basal (time 0; pre-dextrose injection) insulin levels in GRMD (mean 31.26 μIU/ml; *p* < 0.05) compared with combined normal/carrier groups (mean 21.61 μIU/ml; *p* < 0.05) (Fig. 4b). The GRMD insulin levels peaked at the 5-min time point, while both carrier and normal dogs did not peak until 15 min (Fig. 4b).

### PET/CT: Skeletal Muscle

**Fig. 5:**
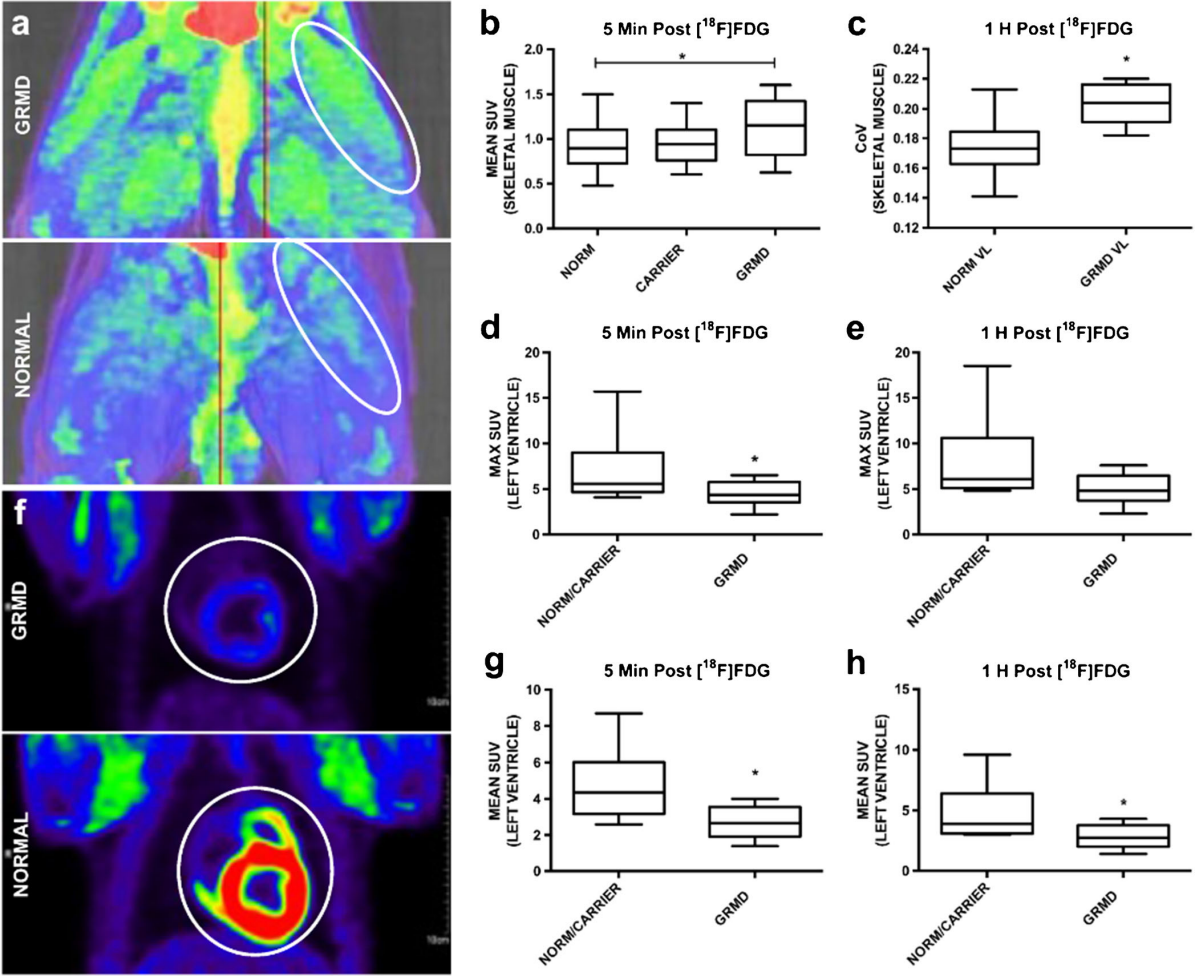
Differential [^18^F]FDG uptake with PET/CT in GRMD skeletal and cardiac muscle. **a** GRMD VL, rectus femoris, and CS (top, white oval) had higher SUV than normal littermates (bottom; white oval). **b** Skeletal muscle had increased mean SUV at 5 min post-[^18^F]FDG/insulin in GRMD compared with normal. **c** GRMD skeletal muscle had significantly higher CoV at 1 h. In cardiac muscle, **d** SUVmax and **g** SUVmean were lower in the GRMD **f** left ventricle (top) compared with normal (bottom) and carrier at 5 min post-[^18^F]FDG/insulin (white circle outlining the heart). At 1 h, **e** SUVmax trended and **h** SUVmean were significantly lower in GRMD compared with normal left ventricle. *p < 0.05.

Based on the increased GLUT-4 membrane localization and initial rapid uptake of BG, we hypothesized that *in vivo* imaging with positron emission tomograhy (PET)/X-ray computed tomography (CT) would be able to detect more rapid glucose uptake in GRMD skeletal muscle compared with normal/carrier dogs. With administration of 2-deoxy-2-[^18^F]fluoro-d-glucose ([^18^F]FDG), a glucose analog, and co-administraion of insulin, genotype significantly increased mean skeletal muscle SUV at 5 min post-injection scan (*p* = 0.03) (Fig. 5a, b); this difference was lost at the 1-h time point. The mean SUV was significantly different among the CS, VL, and rectus femoris muscles at both time points (*p* < 0.01), regardless of genotype. Max SUV was not significantly different between muscles, genotype, or scan time.

To normalize for limb bloodflow, we assessed SUV in a nearby tissue (pelvic fat) that should not be influenced by disease status. Mean SUV in a 1-cm ROI of pelvic fat showed no significant differences between the groups at either time point; however, max SUV was significantly higher in normal dog pelvic fat (*p* = 0.03). There was no significant differences in systolic, diastolic, and mean blood pressure between groups at the time points before, just after, and 1 h after [^18^F]FDG administration (Suppl. Table 1).

We hypothesized that severely affected muscles with heterogeneous muscle composition due to segmental necrosis, including areas of fibrosis, inflammation, degeneration, and myofiber regeneration would have more heterogeneous [^18^F]FDG uptake. Using CoV (SD SUV/mean SUV) as a marker for heterogeneous uptake, values were significantly higher in GRMD VL muscle (CoV = 0.204; *p* = 0.02) *vs.* normal dogs (CoV = 0.174) at the 1-h scan (Fig. 5c). Average CoV for carriers was midway between the GRMD and normal averages (CoV = 0.181; *p* = 0.29), although significance was not reached. The CoV of the hypertrophied CS muscle, which is relatively spared with minimal pathology (inflammation, degeneration) [20], did not differ between GRMD and normal/carrier dogs.

### PET/CT: Cardiac Muscle

Based on preferential involvement of the posterobasal heart region in DMD histopathologically and in previous imaging studies [22–24], we hypothesized that [^18^F]FDG uptake would be increased in these areas in GRMD. At the 5-min post-[^18^F]FDG /insulin scan, both mean (2.7 *vs.* 4.8; *p* = 0.007) and max (4.5 *vs.* 7.1; *p* = 0.04) SUV were suprisingly *lower* in GRMD *vs.* carrier and normal hearts (Fig. 5d–h). At 1-h post-[^18^F]FDG administration, mean SUV (2.8 *vs.* 4.7; *p* = 0.02) was still significantly lower in GRMD, though the difference in max SUV (4.9 *vs.* 7.8; *p* = 0.06) was no longer significant. Mean and max SUV did not differ among the standard 16 LV segments or with CoV in the groups (*p* > 0.3).

## Discussion

In addition to the mechanical fragility of the sarcolemma, dystrophin deficiency is characterized by metabolic dysregulation, manifesting as reduced glycolytic enzymes, mitochondrial structural and functional abnormalities, and altered glucose uptake and response to insulin [4, 8, 11, 19, 25–28]. Reduced muscle mass and increased fat are risk factors and could contribute to altered insulin sensitivity, independent of dystrophin. However, the presence of hyperglycemia and hypoglycemia in boys with normal or low body mass index [11] suggests that the dystrophy state, and not just body composition, alters insulin sensitivity in DMD. Cumulatively, these changes lead to a “metabolic crisis” in dystrophin deficient myofibers, which could result in greater susceptibility to ischemia, metabolic stress, and reduced regenerative capacity.

Glucose transport into muscle cells in response to insulin or contraction [29] occurs primarily through translocation of GLUT4 from the cytoplasm to the sarcolemma/T-tubules [10]. As such, alterations in GLUT4 levels or trafficking within the cell significantly impact overall glucose metabolism. Changes in GLUT4 localization has been noted in both DMD and mdx muscles, but it is unclear if this altered trafficking is a direct result of dystrophin loss or a secondary response to metabolic stress. We hypothesized that GLUT4 alterations in GRMD would be similar to DMD muscle [11], contributing to the metabolic dysregulation in dystrophic muscle.

We queried microarray profiles and found reduced GLUT4 mRNA in dystrophin-deficient muscles. However, our follow-up studies showed varying protein levels, being similar to normal dogs in GRMD CS and VL muscles, but increased in GRMD LDE. The protein results in the CS and VL are in keeping with unchanged levels of GLUT4 protein expression in type II diabetes, though mRNA is also unchanged in that disease [30, 31]. On the other hand, increased levels in GRMD LDE were consistent with elevated values in hind limb muscles of the mdx mouse, regardless of age [12]. Interestingly, GRMD LDE had the most profound changes in GLUT4 mRNA and protein, suggesting a negative feedback loop may be involved between mRNA and protein. Moreover, these molecular changes in GRMD LDE may be due to the severe dystrophic phenotype and significant degeneration/regeneration observed in this muscle (LDE is atrophied/wasted to half the size of normal) [32]. With regard to the left ventricle of the heart, there was a trend for increased GLUT-4 in GRMD (*p* = 0.06). Perhaps with the availability of more samples, statistical significance could be achieved.

While overall GLUT4 protein levels in the tissues were not increased in most GRMD muscles evaluated, immunofluorescence microscopy surprisingly revealed increased GLUT4 localization at the myofiber cell membrane in all GRMD skeletal muscle evaluated. This membrane localization could represent a dysregulation in cellular trafficking or a physiological response to increased metabolic demand for glucose (due to the metabolic crisis). On that note, GLUT4 translocation to the muscle membrane in this model may represent a sort of “priming” to allow for rapid extracellular glucose uptake. Interestingly, normal and GRMD muscles had similar amounts of cytoplasmic GLUT4 aggregates, representing a potential therapeutic reservoir for the latter. With regard to cardiomyocytes, we did not detect a statistical difference in GLUT4 membrane expression between normal and GRMD left ventricle samples. Lesions and associated clinical disease occur much earlier in skeletal *vs.* cardiac muscle in DMD and GRMD, raising questions of factors that could either accelerate skeletal muscle or delay cardiac involvement. These alterations in skeletal *vs.* cardiac muscle GLUT4 translocation may represent the differences in severity between the two tissues.

Although we did not co-stain for GLUT-4 and fiber type, it should be noted that most dystrophin-deficient myofibers undergo fiber type switching from fast to slow twitch [4]. We can then infer that GRMD myofibers with GLUT-4 membranous expression were of the slow twitch phenotype. A previous study in human muscle showed small, but significant differences in GLUT-4 expression and fiber type; however, the authors concluded that GLUT-4 protein content was related more closely to activity level than fiber type [33].

We recently revealed that the morphologically spared and hypertrophied GRMD CS muscle had a reduction in expression in several glycolytic enzymes, including phosphoglucomutase-1, 6-phosphofructokinase, and glucose-6-phosphate isomerase [4]. In the current study, we evaluated at the mRNA level, HK-1, the first enzyme of the glycolytic pathway, and observed an increased expression in GRMD LDE muscle. These differences in glycolytic enzyme expression may be due to muscle specific changes that occur between hypertrophied (CS) and atrophied (LDE) muscle in GRMD and should be further explored. Nevertheless, the increased HK1 is in keeping with our hypothesized compensatory mechanism within dystrophic muscle to rapidly metabolize glucose.

We then hypothesized that increased sarcolemmal GLUT4 could lead to rapid and immediate glucose uptake into skeletal muscle. Indeed, GRMD dogs had rapid BG uptake with a comcomitant insulin peak at 5-min post-dextrose challenge. In contrast, normal/carrier insulin levels peaked at 15 min. This truncated insulin response shows that the apparently rapid glucose uptake was not due to a larger overall insulin level in GRMD. Interestingly, GRMD dogs had higher basal (pre-dextrose injection) BG and insulin levels, suggesting dystrophic muscle is under higher metabolic demand and requires a slightly higher level of BG and insulin levels to compensate. Area under the BG curves did not differ between GRMD and normal/carrier dogs, perhaps because of our failure to sample frequently over the first 15 min and continue sampling for 3 h [34]. Had we employed more frequent early sampling, the slope of GRMD curves might have been increased. In addition, because glucose metabolism varies with age, GRMD and normal/carrier dogs should be matched more closely in future studies. Here, we tested the response to insulin-mediated GLUT4 uptake of glucose. Ideally, contraction-mediated GLUT4 uptake should be tested, but performing a treadmill exhaustion protocol in GRMD dogs, as seen in mice,would not be feasible [35].

We further hypothesized that GLUT-4 localization at the dystrophic myofiber membrane would produce a measurable increase in the immediate uptake of [^18^F]FDG. In order to force rapid and specific uptake into skeletal muscle, we co-administered insulin and [^18^F]FDG tracer at the initiation of scanning. Similar to our IV-GTT results, [^18^F]FDG uptake was higher in GRMD *vs.* normal/carrier dogs at 5 min post-[^18^F]FDG/insulin administration but not at 1 h, consistent with an early, transient response [36]. Most likely, this early transient response to glucose (and a glucose analog) partially compensates for metabolic dysregulation in dystrophic muscle. Other methods such as dynamic PET after [^18^F]FDG administration might demonstrate differences in uptake over time [37]. With regards to exercise, all dogs are provided daily enrichment, including exercise (walking and running outside for a period of time). Normal animals are inherently more active than GRMD dogs, but regular conditioning would be expected to create increased insulin sensitivity in normal muscle (*i.e.*, increasing glucose and glucose analog uptake). For these imaging studies, exercise was minimized in all groups the morning prior to the PET/CT studies to reduce any exercise-related short-term effects on [^18^F]FDG uptake. Nevertheless, our findings in GRMD dogs that increased [^18^F]FDG and dextrose uptake at 5 min post-administration provides a potential biomarker “window” to assess treatments intended to improve muscle metabolism.

Like skeletal muscle, GLUT4 is the major transporter of glucose into cardiomyocytes [38, 39]. Surprisingly, mean and max SUVs were lower on PET studies of the left ventricle in GRMD *vs.* normal/carrier dogs. Since this reduction persisted beyond the 5-min time point, this likely did not occur simply because of selective skeletal muscle uptake. Instead, this presumably reflects a primary cardiac insulin resistance associated with dystrophic cardiomyopathy [16]. While we further hypothesized that [^18^F]FDG distribution would correspond to the regional nature of lesions within the dystrophic heart, mean or max SUV did not vary among the 16 LV segments nor did CoV differ between genotypes. Similarly, GLUT4 protein expression and translocation differences could not be confirmed in the heart, though there was a trend toward increased GLUT4 protein expression in GRMD, which appeared to be affected by higher levels in older dogs. Indeed, further studies in GRMD heart muscle are needed to confirm GLUT-4 trafficking abnormalities due to potential aberrant insulin and/or contraction stimulation.

Taken together, these findings reiterate that glucose dynamics and metabolism vary between cardiac and skeletal muscle. Indeed, these two muscle cell types have different GLUT4 vesicle populations with varying responses to insulin and contraction [38, 39]. This metabolic dissimilarity could also help explain the difference in disease progression between these two tissues, as suggested by recent gene microarray studies in GRMD dogs [40]. Most affected dogs from the PET/CT study are thriving in the colony, which has precluded assessment of GLUT4 expression in their hearts to better clarify these differences.

Blood flow and inflammation can be contributors to differences in [^18^F]FDG uptake, particularly in early PET scans. Due to prolonged washout times and concerns about prolonged anesthesia in the dogs, as well as not wanting to administer an additional tracer to confound results, we did not assess blood flow or inflammation directly in this study. However, blood pressure, which is measured in the pelvic limb, was not significantly different between groups before, during, or after [^18^F]FDG and insulin administration. Additionally, SUV measurements of pelvic fat, which should not be affected by the GLUT4 differences observed in the skeletal muscle between genotypes, did not show significant differences in mean SUV. However, pelvic fat max SUV was higher in normal dogs compared with other groups. Overall, these results suggest that altered blood flow was not a major contributor to increased skeletal muscle SUV in GRMD dogs.

Likewise, we assessed multiple GRMD muscles in the thigh which naturally show differences in degree of pathology, including inflammation. These include the CS, which is hypertrophied but has minimal inflammation, and the VL and rectus femoris that show classic dystrophic changes of inflammation/degeneration [20, 32]. Our statistical analysis revealed that GRMD skeletal muscle had a significant increase in mean SUV at 5 min post-[^18^F]FDG/insulin, but the GRMD genotype did not influence uptake between the individual muscles (CS, VL, rectus femoris). Elevated blood glucose from feeding has been shown to interfere with [^18^F]DG uptake, while co-administration of insulin results in a rapid uptake of 85–90 % of glucose within the first 5 min [41]. Additionally, although inflammatory cells have insulin responsive GLUT4 translocation [42], the inflammatory infiltrate present in GRMD muscle would be expected to interfere with insulin sensitivity, rather than increasing uptake [43]. Therefore, we hypothesize that the primary driver of increased SUV in GRMD skeletal muscle was due to insulin-stimulated GLUT-4 uptake of [^18^F]DG. We acknowledge that further studies should be performed to co-administer [^18^F]FDG, a blood flow tracer, and an inflammatory marker in GRMD dogs to further clarify this issue.

Another potential confounding factor is the age differences between the molecular and PET studies. For our initial molecular assessment of mRNA, protein expression, and qPCR, we used a cohort of frozen samples banked from previous biopsies and muscle sampling at disease-specific time points. When we moved to *in vivo* imaging, we utilized breeder dogs with a wider age range that were currently available and attempted to best match between the examined groups. It is possible that the gene expression and GLUT4 localization profiles in older, affected dogs are different than those seen in younger animals. However, disease differences are manifested and stratified by 6 months of age, and would be expected to be similar or worse in older affected dogs.

Gender is also a potential confounding factor. In people, there are differences in GLUT4 expression and insulin resistance between males and females [41]. Carrier animals in this study were female, while GRMD and normal animals were of mixed gender. Female dogs in diestrus or pregnancy can have increased insulin resistance and higher insulin, but we did not include females which had recently been in heat in this study. However, analysis of glucose or insulin levels stratified by gender did not show a significant difference between genders in our cohort. Previous canine studies of circulating monocyte glucose transporters did not show a difference between gender [42]. We have not detected any functional differences between genders in GRMD dogs, either [44]. As such, we believe sex differences were not a major confounder in this study.

## Conclusions

Overall, our results suggest that glucose handling and metabolism differs between GRMD and normal/carrier dogs. In particular, GRMD dogs have higher levels of both resting insulin and glucose, more rapid glucose uptake, which peaked at different levels compared to normal dogs. We theorize that increased GLUT4 at the cell membrane primes GRMD muscle to induce rapid glucose uptake. These data suggest that glucose metabolism, specifically GTT and PET/CT testing, may be utilized as surrogate biomarkers to assess disease progression and normalization of muscle metabolism in GRMD dogs following various treatments. Further studies are needed to determine if GRMD cardiac cells are more or less insulin responsive than normal. Future imaging studies to evaluate tracers of inflammation, blood flow and fatty acid metabolism in the heart/skeletal muscle would further resolve some of the remaining questions regarding confounding factors of [^18^F]FDG uptake in GRMD.

## Electronic Supplementary Material

ESM 1 (PDF 156 kb)
