# Supplementary material for: Glucose Metabolism as a Pre-clinical Biomarker for the Golden Retriever Model of Duchenne Muscular Dystrophy
Source: Mol Imaging Biol. 2018 Mar 5;20(5):780–8. doi: 10.1007/s11307-018-1174-2 (PMC6153676; doi:10.1007/s11307-018-1174-2)
Supplement: Supplementary file 1 — (PDF 156 kb) [file 11307_2018_1174_MOESM1_ESM.pdf]

Electronic Supplementary Material

Glucose Metabolism as a Pre-Clinical Biomarker for the Golden Retriever Model of Duchenne Muscular Dystrophy

Journal: Molecular Imaging and Biology

Sarah Morar Schneider[1], Vidya Sridhar[2], Amanda K Bettis[3], Heather Heath-Barnett[3], Cynthia J Balog-Alvarez[3], Lee-Jae Guo[2,3], Rachel Johnson[2], Scott Jaques[4], Stanislav Vitha[5], Alan C Glowcwski[2], Joe N Kornegay[3], Peter P Nghiem[3]*

Department of [1]Veterinary Pathobiology, [2]Texas A&M Institute for Preclinical Studies,[3]Department of Veterinary Integrative Biosciences, [4]Texas A&M Veterinary Diagnostic Laboratory, [5]Microscopy Imaging Center, College of Veterinary Medicine and Biomedical Sciences;, Texas A&M University, College Station, TX 77843-4458, United States

Corresponding Author: Peter P. Nghiem, DVM, PhD

Assistant Professor

Department of Veterinary Integrative Biosciences

College of Veterinary Medicine and Biomedical Sciences

Texas A&M University

4458 TAMU

College Station, TX 77843-4458

Phone: (979) 862-9118

Fax: (979) 458-5912

Email: pnghiem@cvm.tamu.edu

Supplemental Methods:

Animals: The details of immunostaining are provided in the electronic supplementary material (ESM). The dogs used in this study were part of a colony carrying the GRMD mutation. Animal care was governed by Texas A&M animal use protocol 2015-0110 (Standard Operating Procedures-Canine X-Linked Muscular Dystrophy) and principles outlined in the National Research Council's Guide for the Care and Use of Laboratory Animals. For the glucose tolerance testing (GTT) and PET-CT scans, we used 6 GRMD dogs (heterozygous male-4, homozygous female-2; 8 months [mo.] to 6 years (y) of age); 6 GRMD carriers (dystrophin +/-; 8mo to 6y) and 6 normal dogs (M-4, F-2, 6 mo to 2 y). Dogs were fed LabDiet Advanced Protocol High Density Canine dry or wet food (PMI Nutrition, St. Louis, MO).

MRNA Profiling: Quantification and analysis methods have been described previously [1, 2]. We queried GLUT4 mRNA expression from previously performed genome-wide mRNA profiles from normal and GRMD dogs at 4-9 weeks and 6 mo. of age in the cranial sartorius (CS), long digital extensor (LDE), and vastus lateralis (VL) muscles. One probe set—Cfa.3539.1.S1_s_at— was identified for GLUT4.

RNA isolation and quantification: Total cellular RNA was isolated with Tripure reagent (Roche, Indianapolis, IN) from frozen skeletal muscle (LDE) and DNase treated with DNA-free kit (Applied Biosystems, Foster City, CA). The RNA concentrations in the individual samples were quantified

using a Nanodrop 2000 spectrophotometer and assessed for quality on a 2100 Bioanalyzer (Agilent Technologies, Santa Clara, CA). The 16 RIN values ranged from 8.0-9.0.

Quantitative PCR analyses: Quantitative RT-PCR (qPCR) primers were designed for genes of interest (GLUT4, hexokimase-1 [HK1]) using Primer Express 3.0 software (Applied Biosystems, Foster City, CA). RNA samples from the microarrayed skeletal muscle (LDE) [1, 2] were DNase treated and reverse transcribed with oligo-dT, random primers, and Superscript II (Invitrogen, Carlsbad, CA). The reverse transcription reactions consisted of 100ng of DNase treated RNA in a 50 ul reaction, ultra pure H_20, oligo dt (2.5ul of 500ng/ul) and random hexamer (0.48ul of 1mM stock) heated to 65 degrees for 5 min and cooled to room temperature. The remaining reagents: Superscript II (2ul), 5X 1st Strand buffer (10ul), 0.1M DTT (5ul), 10mM dNTPs (2.5ul) and an RNase block Ambion's Superasin (1ul). All were heated to 37 ^{o}C for one hour and terminated by heating at 90 ^{o}C. for 5 min. QRT-PCR was performed in triplicate reactions with Power Sybr Green Master Mix. All assays performed on a 7900HT FastStart Real-Time PCR System (Applied Biosystems). Reactions were loaded on a MicroAmp Fast Optical 384 well plate (Applied Biosystems) and consisted of Power Sybr Green, 300 nM of each forward and reverse primer, PCR grade water, and 0.5ul of each reverse transcription reaction (cDNA). The cycling parameters on the 7900HT machine were; 50 ^{o}C for 2 min, 95 ^{o}C for 10 min, and 40 cycling repeats of 95 ^{o}C for 15 s and 60 ^{o}C for 1 min with a dissociation curve added to validate primers.

Western blotting: Protein extraction, quantification, and analysis methods were described previously [4]. GLUT4 antibody (ab65267; Abcam; Cambridge, UK) was incubated with the PVDF

membrane at 1:1,000 dilution in 5% BSA overnight at 4°C. Total protein on the pre-blotted PVDF membrane were used as the loading control to normalize GLUT-4 protein values [3, 4]. Secondary rabbit anti-mouse IgG horseradish peroxidase antibody from Abcam (ab6728) was diluted to 1:5,000 in 5% BSA.

Confocal (immunofluorescence) microscopy: Sample preparation was described previously [1]. Muscle samples were taken by surgical biopsy or at necropsy from the LDE, CS, VL, cranial tibial, diaphragm, and left ventricle of the heart; muscles from a combination of seven normal and nine GRMD samples were analyzed. Samples were imaged with an Olympus (Waltham, MA) FV1000 laser scanning confocal microscope using 20x/0.85 and 100x/1.4 oil immersion objectives. Images were examined with Olympus Fluoview FV1000 software. GLUT4 antibody (ab65267), Anti-SPTBN1 (ab72239), and goat anti-mouse IgG H&L (Alexa Fluor 488; ab 150113) were from Abcam.

ImageJ (NIH; Bethesda, MD) 1.47v software was used for quantification of GLUT4 expression at the myofiber membrane in GRMD and normal muscle samples. Bitmap images taken at 20X objective were uploaded. For skeletal muscle analysis, a software-enabled standard default threshold was set for each image in color red with a dark background. The unit of length was set to “micron”. Fluorescent particles were analyzed with measurements ranging from 50 $microns^2$ to infinity. The area and mean fluorescence of all particles were summed and the normalized fluorescence was calculated per image (summed area/summed fluorescence). Normalized values for all GRMD and normal muscle images were compared. Since cardiac muscle fibers differs in morphology, we could not use the same methodology as for skeletal muscle. As such,

we measured the mean and peak intensity of GLUT-4 signal across the cardiomyocyte membrane. An average of 10 cells per image for the mean and peak intensities were used per sample. GRMD was then compared to normal.

Intravenous (IV) glucose tolerance test (GTT): Dogs were fasted for 15 h prior to testing. Area under curve was calculated for glucose and insulin, as described previously [5]. Weights were taken the day of the procedure and catheters placed in both cephalic veins. After baseline glucose and insulin testing (0 min), each dog received a bolus of 50% dextrose solution intravenously (IV) at 1 g/kg body weight over 30 s. The opposite catheter was used for blood sampling. Three ml of blood were collected at 0 (pre-dextrose administration), 5, 15, 30, 45, 60, and 120 min post-dextrose administration. The samples were centrifuged within 30 min and the serum frozen at -80 ^{o}C for later analysis. Blood glucose (BG) was measured with a Gluco-quant Glucose/HK kit (Roche Diagnostics, Mannheim, Germany) on a AU480 analyzer (Beckman-Coulter, Brea, California). Blood insulin was measured by a radioimmunoassay kit (EMD Millipore Corp., St Charles, Missouri) on an ISO Data 20/20.

Positron emission tomography-computed tomography (PET-CT) acquisition: PET-CT and GTT testing were completed on separate days. Dogs were fasted overnight and kept in a quiet transport cage prior to their scan to minimize excitement, which could influence glucose analogue uptake. Dogs were premedicated (intramuscular acepromazine 0.02 mg/kg, butorphanol 0.4 mg/kg, and atropine 0.04 mg/kg) and catheters were placed in both cephalic veins. Affected and carrier dogs were induced with sevoflurane administered via inhalation;

normal dogs were induced with IV Propofol (5.5 mg/kg, slowly to effect). All dogs were intubated and maintained on Sevoflurane with mechanical ventilation. Intraoperative SpO2, heart rate, and blood pressure were monitored with an Advisor vital signs monitor (SurgiVet, Norwell, MA) and a cuff on the rear limb continuously, with values recorded at 10 min intervals.

Blood glucose was measured after anesthetic induction, just prior to IV administration of the radionuclide 2-deoxy-2-[^{18}F]fluoro-D-glucose ([^{18}F]FDG) (0.05 to 0.1 mCi/kg of body weight), a glucose analogue shown to behave similarly to endogenous glucose. Insulin (0.05 U/kg) was administered IV concurrently with [^{18}F]FDG to encourage intracellular uptake of the radionuclide [6]. Blood glucose was checked every 30 min. We took precautions to keep BG in the normal range (80-210 mg/dl) to prevent hypoglycemia, but also to prevent hyperglycemia, as the latter has been shown to interfere with radionuclide uptake. As such, BG levels were maintained between 70-100 mg/dl. A 10ml bolus of 50% dextrose was given IV if BG dropped below 80 mg/dl.

The PET-CT procedure was performed on a 128-slice Siemens Biograph PET/CT scanner (Siemens Medical Solutions USA, Inc., Malvern, Pennsylvania). Dogs were placed in sternal recumbence with the pelvic limbs extended behind them. They were scanned caudal to cranial beginning two-thirds down the tibia (closer to the hock than the stifle) and progressing to the head with ~20 cm steps, 5 min/step, for seven steps total. A gated cardiac scan of the chest followed each full-body scan. The topogram prescription scout for the initial scan and the initial pair of PET scans began 5 min after [^{18}F]FDG and insulin administration, with a second set of scans at 1 h post administration.

Segmentation and analysis: PET-CT images were analyzed using the Inveon Research Workspace (Siemens Medical Solutions USA, Inc., Malvern, Pennsylvania). For the skeletal muscle analysis, the full body scans were reoriented so that the transverse view was perpendicular to the long axis of the femur and the sagittal and dorsal views were parallel to the sagittal and frontal long axes of the femur. Using the CT scout scan, the muscles were manually segmented at every 5th slice, and the whole muscle region of interest (ROI) was interpolated from these slices [7]. As our functional studies suggest that GRMD disease severity was similar between the left and right limbs, one limb from each dog was examined. The mean standard uptake value (SUV) of [^{18}F]FDG, maximum (max) SUV and standard deviation (SD) of SUV were measured for each of the CS, VL, and rectus femoris muscles at both 5 min and 1 h post-insulin/[^{18}F]FDG administration.

For the cardiac studies, all measurements were obtained using the 4th bin of the gated cardiac scans. Scans were first reoriented such that the transverse, sagittal, and dorsal planes corresponded to the short axis, horizontal long axis (4 chamber), and the vertical long axis (2 chamber) views, respectively. The hearts were analyzed with three methods: 1) a single spherical ROI was placed over the whole left ventricle (LV) to record the max SUV; 2) the LV wall ROI was drawn: the window for PET was adjusted until the view showed an even uptake in the LV, then the slices in the LV were counted on the vertical long axis view; hearts ranged from 59 to 65 slices. Segmentation excluded the apical 5 slices and the first 10 basal slices to avoid overlapping structures that might otherwise alter results. Mean SUV, max SUV, and SD of SUV were recorded for each dog. To obtain a heterogeneity index, coefficient of variance (CoV) was calculated by dividing SD SUV by mean SUV [8]; and 3) to examine regional wall segments

within the LV, 16 individual ROI's within each heart were examined [9, 10]. The 16 ROIs were obtained by segmenting representative basal, mid-cavity, and apical slices for each heart as described in the American Heart Association scientific statement on standardized myocardial segmentation [11]; the true apex could not be reliably identified so was not evaluated.

Statistics: Statistics were performed in both Excel for Mac (Microsoft Corporation, Redmond, WA) and confirmed in Prism software version 6 (GraphPad Software, La Jolla, CA). The Prism software was also used for schematic graph generation. Two-way ANOVAs were employed to test significance for mean and max SUV between genotypes (GRMD, carrier, and normal) and muscle (CS, VL, LDE). ANOVAs were also used to look at differences in SUV for the regional segments in the heart. Non-parametric t-tests (Wilcoxon rank sum tests) were used to test significance of GLUT4 expression and CoV between GRMD, carrier and normal dogs. Significance was defined as $p < .05$.

Supplemental Table 1: Systolic, Diastolic, and Mean Blood Pressures for dogs before, 5-min post and 1 h Post [^{18}F]FDG/insulin administration. NR = Not recorded.

Dogs	10min Prior to [^{18}F]DG			5 min post [^{18}F]DG administration			1 hour post [^{18}F]DG		
	Syst	Diast	Mean	Syst	Diast	Mean	Syst	Diast	Mean
**GRMD**									
F (8m)	119	45	94	120	54	79	131	62	109
M(2y)	129	48	74	105	43	69	152	70	96
M(6y)	93	31	61	99	35	63	118	45	77
M(4y)	113	85	95	102	36	60	103	42	68
F(4y)	NR	NR	NR	NR	NR	NR	NR	NR	NR
M(6y)	NR	NR	NR	91	36	60	128	56	83
**Carrier**									
F(2y)	93	33	57	92	34	55	116	52	80
F(6y)	115	44	90	164	82	111	146	77	94
F(2y)	NR	NR	NR	108	41	70	113	53	75
F(8m)	92	36	70	79	34	60	101	43	73
F(4y)	93	47	66	91	48	67	123	67	94
F(6y)	79	33	62	80	32	62	101	55	70
**Normal**									
M(8m)	134	69	103	133	64	97	110	47	81
F(3y)	121	51	78	115	46	79	153	72	103
M(8m)	101	33	63	103	32	40	92	29	55
F(2y)	92	33	58	92	32	62	105	54	75
M(8m)	93	34	65	88	33	64	95	35	54
M(8m)	76	32	55	93	29	55	76	24	54

References:

[1] Nghiem P, Hoffman E, Mittal P et al (2013) Sparing of the dystrophin-deficient cranial sartorius muscle is associated with classical and novel hypertrophy pathways in GRMD dogs. Am J Pathology 183:1411–1424

[2] Kornegay J, Spurney C, Nghiem P et al (2014) Pharmacologic management of Duchenne muscular dystrophy: target identification and preclinical trials. ILAR J 55:119–149

[3] Gassmann M, Grenacher B, Rohde B, Vogel J (2009) Quantifying Western blots: pitfalls of densitometry. Electrophoresis 30:1845–1855 648

[4] Ghosh R, Gilda JE, Gomes AV (2014) The necessity of and strategies for improving confidence in the accuracy of western blots. Expert Rev Proteomic 11:549–560

[5] Tai MM (1994) A mathematical model for the determination of total area under glucose tolerance and other metabolic curves. Diabetes Care 17:152–154

[6] Bengel F, Higuchi T, Javadi M, Lautamäki R (2009) Cardiac positron emission tomography. J Am Coll Cardiol 54:1–15

[7] Fan Z, Wang J, Ahn M, Shiloh-Malawsky Y, Chahin N, Elmore S, Bagnell CR Jr, Wilber K, An H, Lin W, Zhu H, Styner M, Kornegay N (2014) Characteristics of magnetic resonance imaging biomarkers in a natural history study of golden retriever muscular dystrophy. Neuromuscul Disord 24:178–191

[8] Tahara N, Tahara A, Nitta Y, Kodama N, Mizoguchi M, Kaida H, Baba K, Ishibashi M, Hayabuchi N, Narula J, Imaizumi T (2010) Heterogeneous myocardial FDG uptake and the disease activity in cardiac sarcoidosis. JACC Cardiovasc Imaging 3:1219– 1228

[9] Kemp GJ, Taylor DJ, Dunn SP et al (1993) Cellular energetics of dystrophic muscle. J Neurol Sci 116:201–206 587

[10] Chen Y-W, Zhao P, Borup R, Hoffman E (2000) Expression profiling in the muscular dystrophies: identification of novel aspects of molecular pathophysiology. J Cell Biol 151:1321–1336

[11] Cerqueira MD, Weissman NJ, Dilsizian V et al (2002) Standardized myocardial segmentation and nomenclature for tomographic imaging of the heart. J Cardiovasc Magn Reson 4:203–210
